# Successful R0 resection after chemotherapy, including nivolumab, for gastric cancer with liver metastases: three case reports

**DOI:** 10.1186/s40792-024-01929-3

**Published:** 2024-06-05

**Authors:** Junpei Kawai, Itaru Yasufuku, Masahiro Fukada, Ryuichi Asai, Yuta Sato, Yu Jesse Tajima, Chiemi Saigo, Shigeru Kiyama, Akitaka Makiyama, Yoshihiro Tanaka, Naoki Okumura, Katsutoshi Murase, Tatsuhiko Miyazaki, Nobuhisa Matsuhashi

**Affiliations:** 1 Department of Gastroenterological Surgery and Pediatric Surgery, Gifu University Graduate School of Medicine, 1-1 Yanagido, Gifu, 501-1194 Japan; 2 Department of Pathology, Gifu University Hospital, Gifu University Graduate School of Medicine, 1-1 Yanagido, Gifu, 501-1194 Japan; 3 Cancer Center, Gifu University Hospital, 1-1 Yanagido, Gifu, 501-1194 Japan

**Keywords:** Gastric cancer, Nivolumab, Liver metastases, Conversion surgery, Complete response

## Abstract

**Background:**

Advances in chemotherapy have increased clinical experience with conversion surgery for inoperable advanced gastric cancer. This report describes three patients with unresectable gastric cancer accompanied by multiple liver metastases. In all three patients, nivolumab resolved the liver metastases and subsequent conversion surgery achieved a pathological complete response.

**Case presentation:**

In Case 1, a 68-year-old man with clinical Stage IVB gastric cancer and multiple liver metastases initiated first-line therapy with SOX plus nivolumab. The patient completed 13 cycles; however, only nivolumab was continued for 3 cycles because of adverse events. Distal gastrectomy and partial hepatic resection were performed because of a significant reduction in the size of the liver metastases as observed on magnetic resonance imaging (MRI). In Case 2, a 72-year-old man with clinical Stage IVB gastric cancer and multiple liver metastases initiated first-line therapy with SOX. Because of the subsequent emergence of new liver metastases, the patient transitioned to ramucirumab plus paclitaxel as second-line therapy. Third-line therapy with nivolumab was initiated because of side effects. MRI revealed necrosis within the liver metastasis, and the patient underwent proximal gastrectomy and partial hepatectomy. In Case 3, a 51-year-old woman with clinical Stage IVB gastric cancer accompanied by multiple metastases of the liver and para-aortic lymph nodes began first-line therapy with SOX plus nivolumab. The patient completed 10 cycles; however, only nivolumab was continued for 5 cycles because of adverse events. Computed tomography showed a significant decrease in the size of the para-aortic lymph nodes, while MRI indicated the presence of a singular liver metastasis. Distal gastrectomy and partial hepatic resection were subsequently performed. In all three cases, MRI revealed the presence of liver metastases; however, pathological examination showed no viable tumor cells.

**Conclusions:**

We herein present three cases in which chemotherapy, including nivolumab, elicited a response in patients with multiple unresectable liver metastases, ultimately culminating in R0 resection through conversion surgery. Although MRI showed liver metastases, pathological analysis revealed no cancer, underscoring the beneficial impact of chemotherapy.

## Background

The standard treatment strategy for advanced or recurrent gastric cancer with multiple liver metastases is palliative chemotherapy [1, 2]. Nivolumab has gained recent approval worldwide as a first-line treatment for human epidermal growth factor receptor 2 (HER2)-negative, unresectable gastric cancer [1, 2]. The CheckMate 649 trial demonstrated that adding nivolumab to first-line chemotherapy significantly extended both overall survival- and progression-free survival (PFS) [3]. The ATTRACTION-4 trial also revealed an extension in PFS [4]. Conversion surgery, a new therapeutic approach for advanced gastric cancer, has recently garnered much attention as a potential means to improve patients’ prognosis [5, 6]. Conversion surgery is performed for patients with advanced gastric cancer who exhibit remarkable responses to chemotherapy. Its aim is to achieve complete resection of primary and metastatic lesions if they remain, potentially improving survival outcomes [6]. Few reports have described the complete pathological resolution of liver metastases following chemotherapy in patients with inoperable gastric cancer. We herein describe three patients with gastric cancer with multiple liver metastases in whom chemotherapy combined with nivolumab was effectively employed, resulting in a pathological complete response in the resected liver tissue and facilitating subsequent conversion surgery.

## Case presentation

### Case 1

**Fig. 1:**
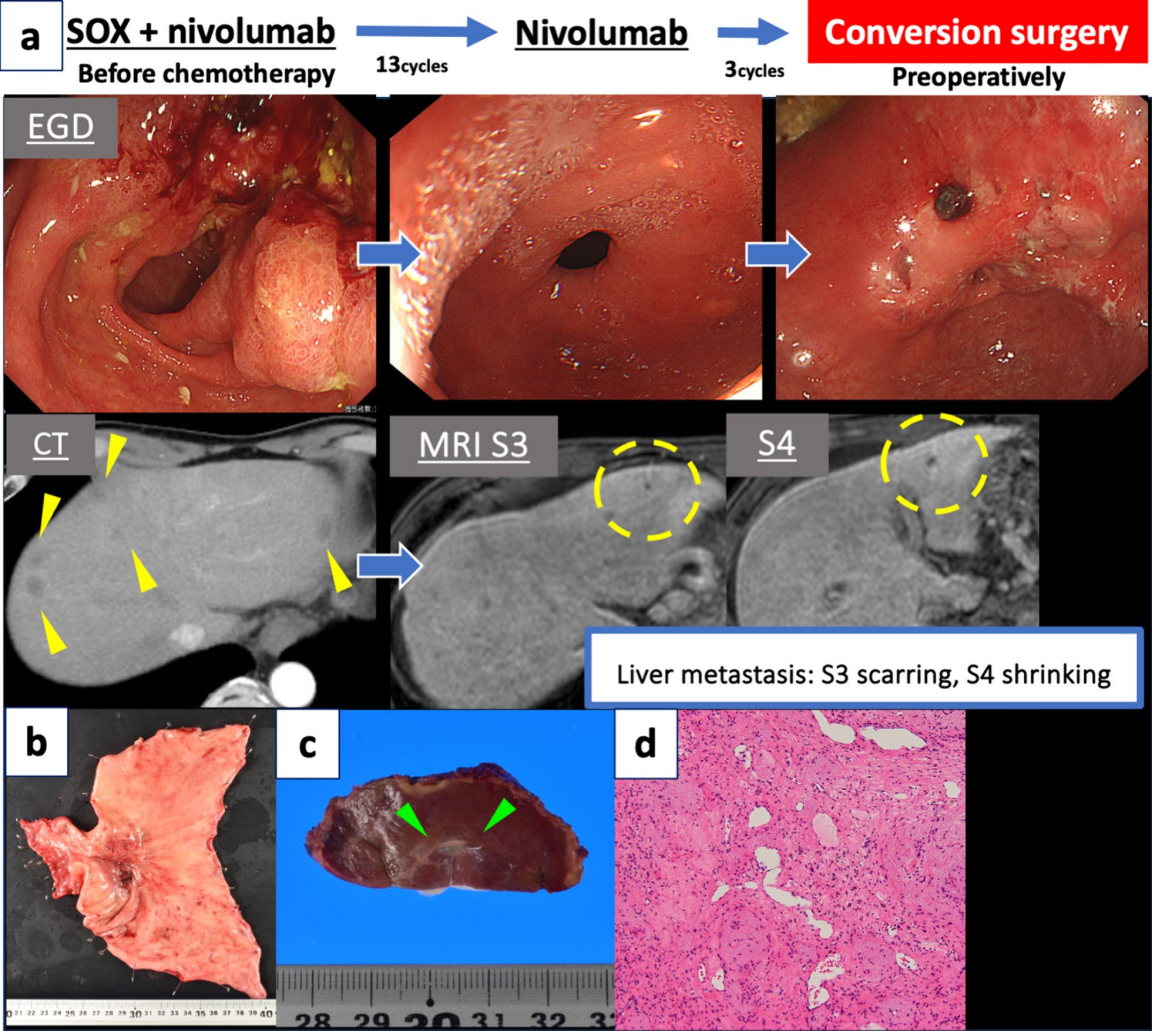
Case 1. **a** Treatment course in Case 1. The patient was diagnosed with advanced gastric cancer and multiple liver metastases (yellow arrowheads) and received SOX plus nivolumab therapy. After 13 cycles of chemotherapy, all liver metastases except those in S3 and S4 were resolved, and the primary lesion was remarkably reduced. MRI showed two remaining liver metastases measuring 5 mm in segment 4 and 3 mm in segment 3 (yellow dashed lines). The patient developed sinus occlusion syndrome caused by oxaliplatin and received three additional cycles of nivolumab monotherapy. The primary lesion slightly increased in size, but the liver metastases remained at a reduced size. The patient thereafter underwent conversion surgery. **b** A Borrmann type 3 tumor was found in the gastric antecubital area, and the pyloric ring was deformed. **c** A white scar was visible on the surface (green arrowheads). **d** The resected liver tissue had transformed into fibrous tissue devoid of malignancies. *EGD* esophagogastroduodenoscopy, *CT* computed tomography, *MRI* magnetic resonance imaging

A 68-year-old man underwent esophagogastroduodenoscopy for epigastric pain, revealing a Borrmann type 3 tumor in the gastric antrum (Fig. 1a). Computed tomography (CT) showed more than multiple liver metastases (Fig. 1a), leading to a diagnosis of clinical Stage IVB [7] gastric cancer. The tumor was negative for HER2, the combined positive score for programmed cell death ligand 1 was > 5, and microsatellite instability was not high. The pre-chemotherapy tumor markers were CEA 46.6 ng/ml and CA19-9 1173 U/ml. The patient initiated first-line therapy with SOX plus nivolumab, receiving 13 cycles while adjusting oxaliplatin administration because of thrombocytopenia, hepatic dysfunction, and peripheral neuropathy.

After completing all 13 cycles of chemotherapy, contrast-enhanced magnetic resonance imaging (MRI) showed two remaining liver metastases measuring 5 mm in segment 4 and 3 mm in segment 3. Most of the liver metastases appeared to have regressed, though the two residual lesions were recognized on MRI (Fig. 1a). Given the substantial reduction in liver metastases on imaging, we considered R0 resection to be feasible and conducted a preoperative assessment.

The assessment revealed that the patient was diagnosed with oxaliplatin and S-1-induced liver damage, and he received three cycles of nivolumab monotherapy. Subsequent examinations showed improved liver damage. The lesion in segment 4 further regressed, and that in segment 3 evolved into a scar. However, the primary tumor necessitated surgery because of progression and outlet obstruction (Fig. 1a). Surgical resection of the primary tumor was planned to control the disease progression. For treatment of the liver metastasis, we planned to resect any scars caused by the tumor, provided that they were present on the surface of the liver, without resection of segments 3 and 4. This decision was based on the MRI findings, which confirmed the need to preserve these segments to assess the pathological response to chemotherapy considering the potential impact on liver function. While tumor markers temporarily normalized, CA19-9 subsequently worsened. Preoperatively, CEA was 4.5 ng/ml, and CA19-9 was 434 U/ml.

Thirteen months after treatment initiation, laparoscopic distal gastrectomy with D2 lymphadenectomy and laparoscopic partial hepatectomy was performed as conversion surgery. Intraoperative contrast-enhanced ultrasonography was not performed during partial hepatectomy because of visible scar tissue. The final diagnosis was L, ant, Type 2, por2 > tub2, ypT4a(SE), Ly1b, V1b, pPM0 (50 mm), pDM0 (35 mm), ypN3b (25/26), and ypStage IIIC [7] (Fig. 1b). The primary tumor exhibited a grade 1a histological response [7]. The resected liver tissue had transformed into fibrous tissue devoid of malignancies and tumor cells (Fig. 1c, d).

The patient received S-1+docetaxel therapy postoperatively. At the time of this writing, he had remained alive for 7 months without relapse of liver metastasis. Both CEA and CA19-9 remained within normal limits. The remaining liver lesions in segments 3 and 4 were being carefully followed up, with plans for secondary surgery if the lesions enlarged without the emergence of new metastatic lesions.

### Case 2

**Fig. 2:**
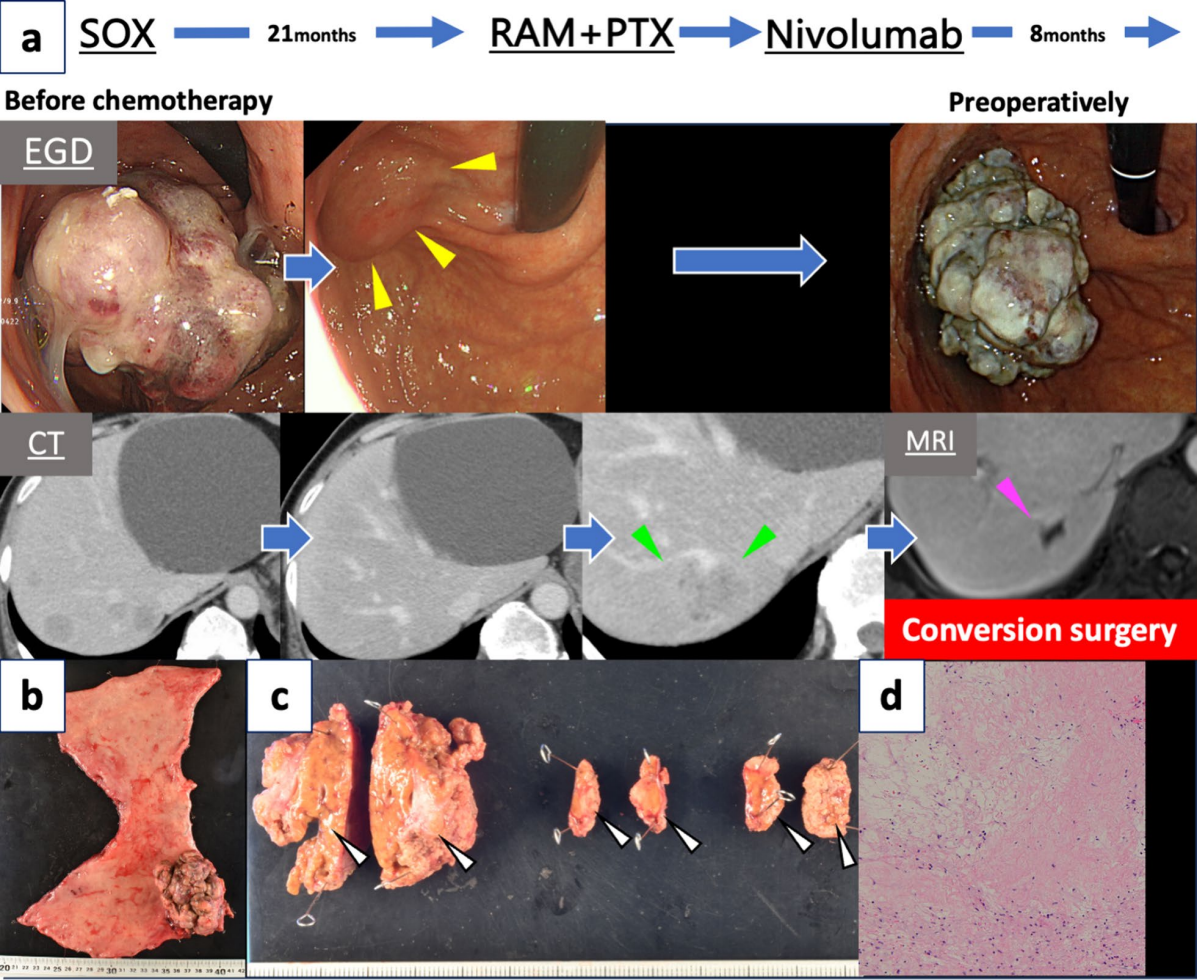
Case 2. **a** Treatment course in Case 2. The patient was diagnosed with advanced gastric cancer and multiple liver metastases and received SOX therapy. The primary lesion shrank (yellow arrowheads) and the liver metastases disappeared soon after the start of treatment. Twenty-one months after starting SOX therapy, the patient was switched to ramucirumab plus paclitaxel therapy because of the appearance of new liver metastases (green arrowheads). Only one cycle of ramucirumab plus paclitaxel therapy was completed because of severe neutropenia, and the patient was then started on nivolumab therapy. Eight months after nivolumab therapy, contrast-enhanced magnetic resonance imaging showed necrosis of the liver metastasis (pink arrowhead). However, the primary lesion continued to grow. Consequently, the patient underwent conversion surgery. **b** A Borrmann type 1 tumor was found on the fornix. **c** White nodules on the cross-section of the liver (white arrowheads). **d** Viable atypical cells disappeared from all specimens. The same area showed fibrosis and edema. *RAM* ramucirumab, *PLX* paclitaxel, *EGD* esophagogastroduodenoscopy, *CT* computed tomography, *MRI* magnetic resonance imaging

The patient was a 71-year-old man with multiple liver metastases from gastric cancer. The primary lesion was a Borrmann type 1 tumor in the fornix, and more than 10 liver metastases were seen on CT imaging (Fig. 2a). HER2 and programmed cell death ligand 1 were both negative. Initially, the patient underwent 23 cycles of first-line SOX therapy at a different medical facility, leading to a near-complete response in the liver metastases. The primary lesion also markedly decreased in size. However, this treatment was stopped when a new liver metastasis appeared in segment 7 (Fig. 2a). Subsequent second-line therapy with ramucirumab and paclitaxel was halted after just one cycle because of severe neutropenia. A third-line regimen was initiated with nivolumab monotherapy. The primary lesion was initially maintained at a reduced size but subsequently showed renewed growth. Contrast-enhanced CT of the liver metastasis in segment 7 showed tumor shrinkage. Contrast-enhanced MRI of the liver metastases revealed that there was an indication of necrosis within the liver metastases (Fig. 2a). No other new metastatic lesions were identified with imaging examinations. Surgical resection of the primary tumor and liver lesions located in segment 7 was planned as conversion surgery. Upon evaluation at our medical facility, the preoperative tumor markers were within normal limits.

At 32 months after initiation of first-line chemotherapy and 8 months after initiation of nivolumab therapy, the patient underwent proximal gastrectomy with D2 lymphadenectomy and partial hepatectomy for segment 7 and another two scarred lesions on the liver surface. A contrast-enhanced intraoperative ultrasound revealed a poorly contrasted area in only segment 7. The final pathological diagnosis was U, post, Type 1, por1, ypT2(MP), INFb, Ly1a, V1b, pPM0 (25 mm), pDM0 (85 mm), ypN0 (0/40), and ypStage IB [7] (Fig. 2b). The primary tumor exhibited a grade 1a histological response [7]. Three resected liver resected were examined, confirming the absence of viable atypical cells post-chemotherapy. Furthermore, tumor cell disappearance was achieved (Fig. 2c, d).

The patient did not receive postoperative chemotherapy. At the time of this writing, he had remained alive without disease relapse for 7 months. CEA and CA19-9 remained within normal limits in the postoperative period.

### Case 3

**Fig. 3:**
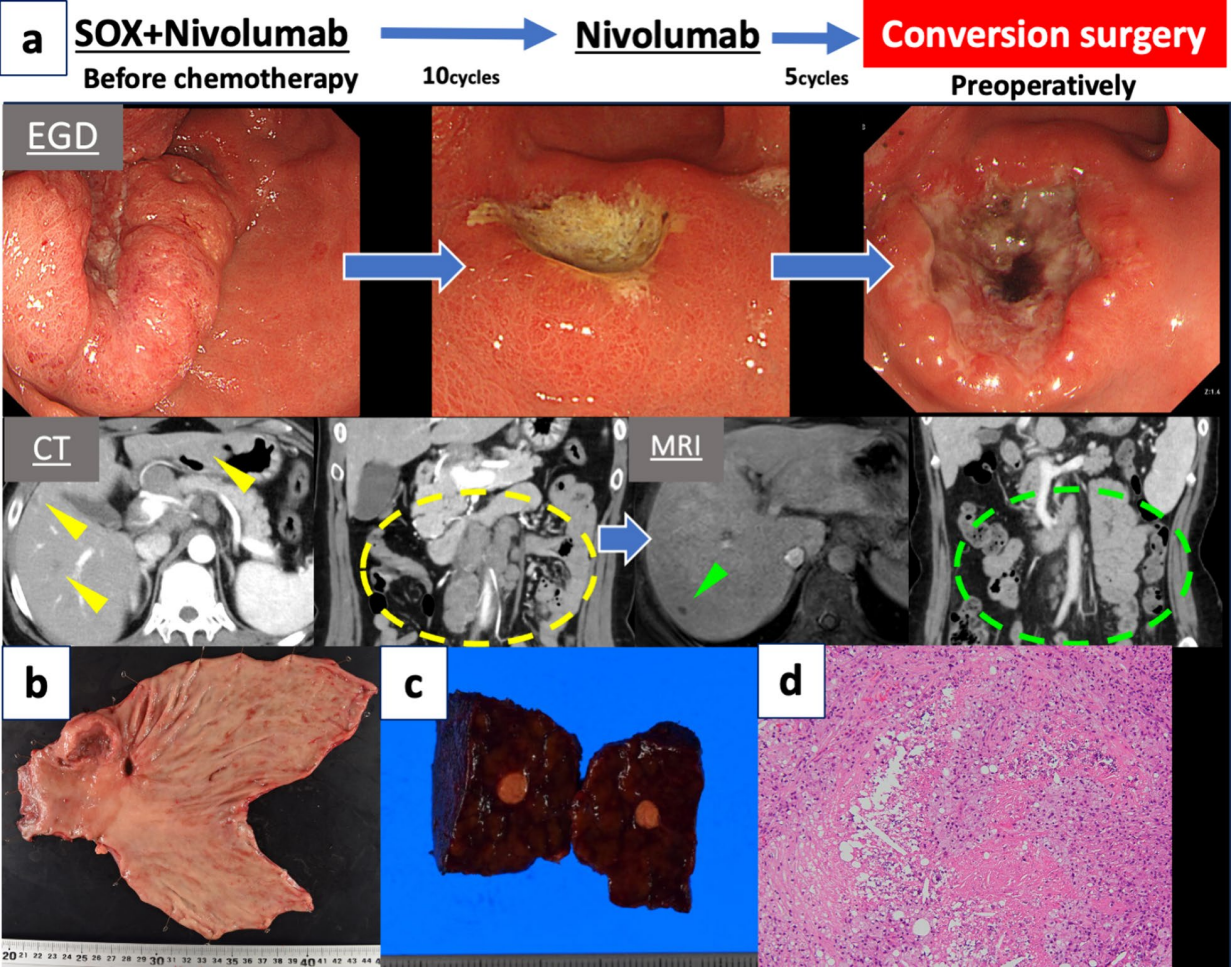
Case 3. **a** Treatment course in Case 3. The patient was diagnosed with advanced gastric cancer, multiple liver metastases (yellow arrowheads), and multiple para-aortic lymph node (PALN) metastases (yellow dashed lines) and received SOX plus nivolumab therapy. After 10 cycles of chemotherapy, all liver metastases (except those in S7) and the PALN metastases were resolved (green arrowhead and dashed lines), and the primary lesion was remarkably reduced. The patient developed sinus occlusion syndrome caused by oxaliplatin and received five additional cycles of nivolumab monotherapy. The primary lesion increased in size, but the liver metastasis and the PALN metastases were maintained at a reduced size. The patient thereafter underwent conversion surgery. **b** A Borrmann type 3 tumor with two ulcers in the gastric lesser curvature and anterior wall. **c** White nodules in the liver. **d** Viable atypical cells disappeared from all specimens. The site contained an inflammatory cell infiltrate and some fibrosis. *EGD* esophagogastroduodenoscopy, *CT* computed tomography, *MRI* magnetic resonance imaging

A 51-year-old woman presented with epigastric pain and elevated tumor marker levels. Esophagogastroduodenoscopy revealed a Borrmann type 3 tumor on the anterior wall of the gastric body (Fig. 3a). CT scans indicated multiple para-aortic lymph node (PALN) metastases, including No. 16b2 [7], and four liver metastases in segments 2, 5, 6, and 8. HER2 was negative, and the combined positive score was ≥ 5. The pre-chemotherapy tumor markers were CEA 46.9 ng/ml and CA19-9 7242 U/ml.

The PALN metastases resolved after 10 cycles of SOX plus nivolumab therapy. MRI showed that most of the liver metastases had regressed, leaving only a 5-mm nodule in segment 7 (Fig. 3a). The patient subsequently received five cycles of nivolumab monotherapy because of chemotherapy-induced liver damage. During this period, the primary tumor tended to increase in size (Fig. 3a), but the disappearance of the PALN metastases and shrinkage of the liver metastases were maintained. Tumor markers mainly were improved, with a preoperative CEA of 0.8 ng/ml and CA19-9 of 74.8 U/ml.

Eleven months after treatment initiation, distal gastrectomy with D2 lymphadenectomy and partial hepatectomy of segment 7 were performed as conversion surgery. The lesion in segment 7 was visualized as a non-contrasting area during intraoperative contrast-enhanced ultrasonography. The final pathological diagnosis was M, ant, Type 3, muc > tub2, ypT2(MP), INFb, Ly1b, V1b, pPM0 (90 mm), pDM0 (25 mm), ypN2(3/36), and ypStage IIB [7] (Fig. 3b). The primary tumor exhibited a grade 1b histological response [7]. The resected liver tissue showed no viable atypical cells, suggesting their elimination through chemotherapy and the achievement of tumor cell disappearance (Fig. 3c, d).

The patient received S-1 plus docetaxel therapy postoperatively. At the time of this writing, she had remained alive without disease relapse for 6 months. Both CEA and CA19-9 remained within normal limits.

## Discussion

The standard approach to managing unresectable advanced or recurrent gastric cancer is palliative chemotherapy [1, 2]. However, the prognosis is often unfavorable, with a median survival time of only 15 months [8, 9]. Liver metastasis is a common occurrence in patients with gastric cancer. Although surgical resection has shown promising outcomes in patients with a single liver metastasis, the role of surgical intervention in patients with multiple liver metastases remains uncertain [10–15]. Shirasu et al. [16] compared the outcomes of surgery-first and chemotherapy-first approaches for patients with two to three liver metastases. Their analysis demonstrated that a chemotherapy-first strategy significantly improved PFS, with subsequent liver resection performed after a positive chemotherapy response in three cases.

Conversion surgery, designed for patients with unresectable clinical Stage IVB [7] gastric cancer who respond to chemotherapy, offers the potential for long-term survival and even cure [5]. The CONVO-GC-1 study, the most extensive international multicenter study to date, retrospectively evaluated 1206 patients with Stage IV gastric cancer who underwent conversion surgery [17]. In that study, achieving R0 resection in conversion surgery for liver metastases resulted in a median survival time of 95.2 months for patients with a single liver metastasis and 56.6 months for those with three or more metastases, demonstrating positive outcomes even for patients with multiple liver metastases [17]. These findings indicate that conversion surgery is a promising approach for treating multiple liver metastases.

Despite the extensive research on liver metastases in patients with gastric cancer, there is a lack of information regarding how frequently cancer cells persist in recognizable liver metastases or the specific imaging characteristics of such cases following chemotherapy. A PubMed search for similar case reports published since 2000, using keywords such as “gastric cancer, liver metastasis, complete response,” “gastric cancer, liver metastasis, complete remission,” and “gastric cancer, liver metastasis, conversion surgery,” yielded 52 reports. Among the 47 reports available since 2007, 41 described patients with gastric cancer in whom liver metastases had responded to chemotherapy, leading to their disappearance or significant reduction in imaging (excluding 6 reports with unclear imaging findings or insufficient laboratory data). In 17 cases, liver metastases were still recognizable on imaging after chemotherapy [18–33]. In eight of these cases, liver resection was deemed necessary, and in seven, pathological evidence confirmed the disappearance of cancer cells. Most of the 17 patients with image-recognized liver metastases achieved long-term survival, but some developed recurrence of liver metastases. The significance of resecting markedly reduced liver metastases as shown by imaging remains uncertain. However, given the importance of achieving R0 resection in conversion surgery [17], hepatic resection may be considered to achieve a potential cure and long-term survival if the liver metastases can be safely excised.

The integration of nivolumab into conventional chemotherapy regimens has resulted in a 10% to 15% increase in response rates [3, 4]. Nivolumab holds promise for reducing or eliminating liver metastases, potentially expanding the pool of patients eligible for conversion surgery. All three of our patients received nivolumab, resulting in the complete eradication of tumor cells and their replacement with fibrous tissue in all liver metastases visible on contrast-enhanced MRI. In the above-mentioned reports of successful chemotherapy for liver metastases of gastric cancer, nivolumab was utilized in six patients [28, 29, 32–35]. Of these, only three underwent conversion surgery for liver metastases. The number of reported cases to date remains small. Analysis of more cases will reveal whether these characteristics are unique to nivolumab and how it compares to chemotherapy alone.

This study has two main limitations. First, we reported only three cases. Second, nivolumab has only been available for a short time to date. If more clinical experience is gained and similar case data can be collected, it may be possible to develop a standard treatment strategy for liver metastasis of gastric cancer.

## Conclusion

In this report, we presented three cases in which multiple unresectable liver metastases at diagnosis responded favorably to chemotherapy, including nivolumab, ultimately leading to R0 resection through conversion surgery. Furthermore, although preoperative imaging indicated the presence of liver metastases in Cases 2 and 3, pathological examination revealed no residual cancer cells in the resected liver metastases, demonstrating the efficacy of the chemotherapy.

## Data Availability

The data can be accessed from PubMed (https://pubmed.ncbi.nlm.nih.gov) or the Japan Medical Abstracts Society (https://search.jamas.or.jp).

## Abbreviations

PALN: Para-aortic lymph node
PFS: Progression-free survival
CT: Computed tomography
HER2: Human epidermal growth factor receptor 2
MRI: Magnetic resonance imaging
